# PD-L1 detection using ^89^Zr-atezolizumab immuno-PET in renal cell carcinoma tumorgrafts from a patient with favorable nivolumab response

**DOI:** 10.1186/s40425-019-0607-z

**Published:** 2019-06-03

**Authors:** Joseph Vento, Aditi Mulgaonkar, Layton Woolford, Kien Nham, Alana Christie, Aditya Bagrodia, Alberto Diaz de Leon, Raquibul Hannan, Isaac Bowman, Renee M. McKay, Payal Kapur, Guiyang Hao, Xiankai Sun, James Brugarolas

**Affiliations:** 1 Kidney Cancer Program, Simmons Comprehensive Cancer Center, University of Texas Southwestern Medical Center, Dallas, TX USA; 2 Department of Internal Medicine, University of Texas Southwestern Medical Center, Dallas, TX USA; 3 Department of Radiology, University of Texas Southwestern Medical Center, Dallas, TX USA; 4 Division of Hematology/Oncology, Department of Internal Medicine, University of Texas Southwestern Medical Center, Dallas, TX USA; 5 Department of Clinical Sciences, University of Texas Southwestern Medical Center, Dallas, TX USA; 6 Department of Urology, University of Texas Southwestern Medical Center, Dallas, TX USA; 7 Department of Radiation Oncology, University of Texas Southwestern Medical Center, Dallas, TX USA; 8 Department of Pathology, University of Texas Southwestern Medical Center, Dallas, TX USA; 9 Advanced Imaging Research Center, University of Texas Southwestern Medical Center, Dallas, TX USA

**Keywords:** Kidney cancer, Renal cancer, Immuno-PET, PD-L1, PD-1, PDX, Patient-derived xenograft, IO, Immunotherapy, Immune checkpoint inhibitors, Predictive biomarkers, SABR, SBRT

## Abstract

**Background:**

Programmed death-ligand 1 (PD-L1) expression in metastatic renal cell carcinoma (RCC) correlates with a worse prognosis, but whether it also predicts responsiveness to anti-PD-1/PD-L1 therapy remains unclear. Most studies of PD-L1 are limited by evaluation in primary rather than metastatic sites, and in biopsy samples, which may not be representative. These limitations may be overcome with immuno–positron emission tomography (iPET), an emerging tool allowing the detection of cell surface proteins with radiolabeled antibodies. Here, we report iPET studies of PD-L1 in a preclinical tumorgraft model of clear cell RCC (ccRCC) from a patient who had a favorable response to anti-PD-1 therapy.

**Case presentation:**

A 49-year-old man underwent a cytoreductive nephrectomy in 2017 of a right kidney tumor invading into the adrenal gland that was metastatic to the lungs and a rib. Histological analyses revealed a ccRCC of ISUP grade 4 with extensive sarcomatoid features. IMDC risk group was poor. Within two hours of surgery, a tumor sample was implanted orthotopically into NOD/SCID mice. Consistent with an aggressive tumor, a renal mass was detected 18 days post-implantation. Histologically, the tumorgraft showed sarcomatoid differentiation and high levels of PD-L1, similar to the patient’s tumor. PD-L1 was evaluated in subsequently transplanted mice using iPET and the results were compared to control mice implanted with a PD-L1-negative tumor. We labeled atezolizumab, an anti-PD-L1 antibody with a mutant Fc, with zirconium-89. iPET revealed significantly higher ^89^Zr-atezolizumab uptake in index than control tumorgrafts. The patient was treated with high-dose IL2 initially, and subsequently with pazopanib, with rapidly progressive disease, but had a durable response with nivolumab.

**Conclusions:**

To our knowledge, this is the first report of non-invasive detection of PD-L1 in renal cancer using molecular imaging. This study supports clinical evaluation of iPET to identify RCC patients with tumors deploying the PD-L1 checkpoint pathway who may be most likely to benefit from PD-1/PD-L1 disrupting drugs.

## Background

We present a patient with poor-risk metastatic ccRCC with sarcomatoid features and high PD-L1 expression whose disease progressed rapidly despite high-dose interleukin 2 (HD-IL2) and pazopanib, and who had a sustained partial response (PR) on nivolumab, as well as corresponding molecular imaging analysis of PD-L1 using immuno-PET in tumorgraft models.

Sarcomatoid differentiation as well as high PD-L1 expression are both associated with aggressive disease [1–4]. Notably, emerging data suggest that sarcomatoid ccRCCs may be particularly responsive to checkpoint inhibitors [5]. Tannir and colleagues conducted retrospective analyses of patients with sarcomatoid tumors from the intermediate/poor-risk cohort in CheckMate-214 (a phase III clinical trial in metastatic ccRCC patients of ipilimumab/nivolumab vs. sunitinib), and found objective response rates of 57% [6].

However, how responsiveness relates to PD-L1 expression in RCC remains unclear. While it seems intuitive that tumors with PD-L1 expression may be engaging this checkpoint pathway, the landmark CheckMate-025 trial found that PD-L1 expression (assessed by immunohistochemistry with a threshold of > 1% of tumor cells) was not predictive of overall survival in patients treated with nivolumab [3]. However, there were limitations including a sampling bias, which is particularly problematic given the well-established intratumoral and metastatic heterogeneity of ccRCC [7].

## Case presentation

### Clinical course

**Fig. 1:**
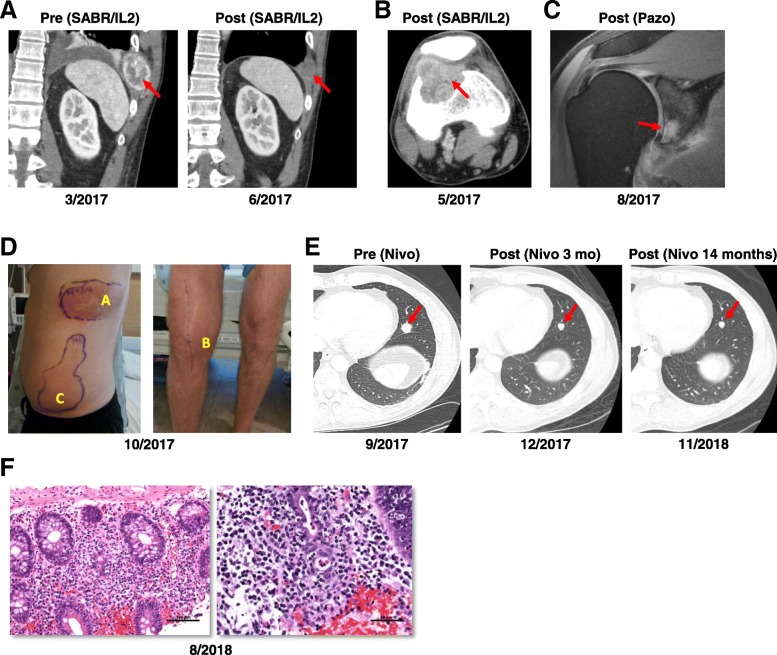
Clinical case. **a** Coronal contrast-enhanced CT images of a lytic metastasis in the left 10th rib (red arrow) before and after SABR and HD-IL2. **b** Axial contrast-enhanced CT image of new lytic metastasis in the right distal anterolateral femur (red arrow), which developed after SABR/HD-IL2 therapy. **c** Coronal proton density fat saturated MR imaging of an osseous metastasis in right glenoid (red arrow) that developed while on pazopanib therapy. **d** Clinical images illustrating radiation recall dermatitis 11 days after first nivolumab infusion at two prior sites of radiation, the left rib (A, radiated six months prior) and the right knee (B, radiated one month prior). Outlined is an area of subcutaneous edema and discoloration (C) attributed to drainage from lesion A. **e** Axial contrast-enhanced CT scan of the chest of representative lingular nodule (red arrow) improving with nivolumab therapy. **f** Hematoxylin and eosin stains of left colon biopsy with increased intraepithelial lymphocytes and cryptitis representative of autoimmune colitis

A 49-year-old man presented in February 2017 with chest wall pain and weight loss, leading to the diagnosis of a ccRCC with metastases to the lungs and a rib (Fig. 1). Based on his presentation with anemia, hypercalcemia, and the need for prompt initiation of systemic therapy, his International Metastatic Renal Cell Carcinoma Database Consortium (IMDC) scoring predicted a poor prognosis with a median survival of 7.8 months [8]. Initial management included a right radical cytoreductive nephrectomy, which required a partial hepatectomy. Pathological analyses showed a 9 cm ccRCC invading into the perirenal and renal sinus adipose tissue as well as the ipsilateral adrenal gland of ISUP grade 4 with extensive sarcomatoid differentiation. Eight out of eight lymph nodes were positive for metastatic disease. Staging was consistent with a pT4N1 tumor. IHC studies showed positivity for CK AE1/AE3, and CA-IX. CK7 was negative. PBRM1 and BAP1 were present suggestive of a wild-type state. PD-L1 was expressed in more than 30% of tumor cells. Given the age of the patient, germline testing was pursued using a CancerNext-Expanded genetic panel including genes such as *VHL, BAP1, FLCN* and *PTEN*, but did not reveal any mutations.

**Fig. 2:**
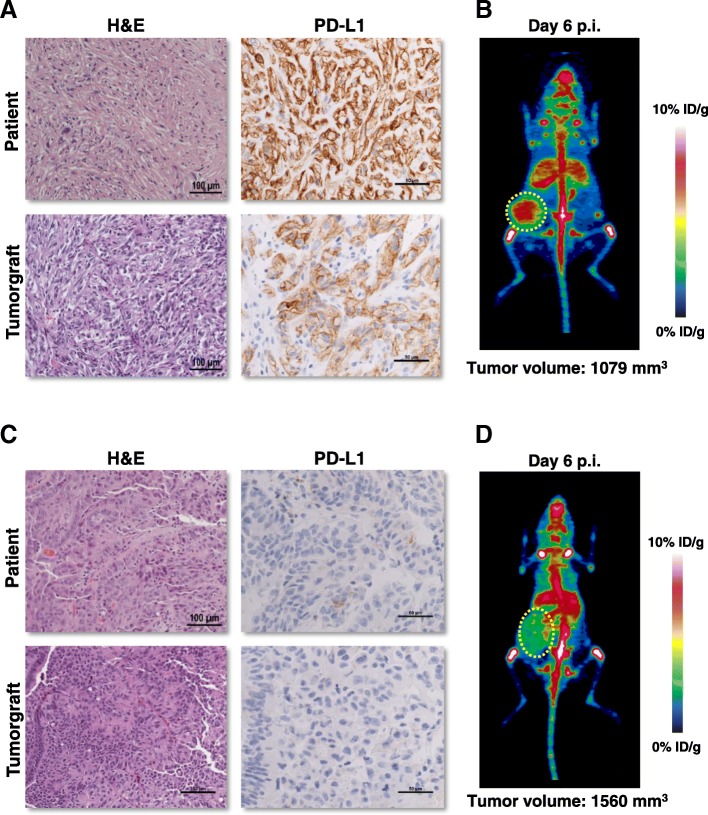
Tumorgraft immunoPET studies. **a** Patient’s tumor (nephrectomy sample) and corresponding tumorgraft demonstrating sarcomatoid differentiation and high PD-L1 expression by IHC. **b** iPET from representative NOD/SCID mouse with subcutaneous tumorgraft. **c**-**d** Images (patient and tumorgraft) from papillary RCC tumor chosen as a control because of low PD-L1 levels. Tumor volumes shown for the individual mice are estimated based on the CT volume quantification of the tumors

Within two hours of surgery, a sample of the patient’s tumor was implanted orthotopically into several NOD/SCID immunocompromised mice to generate a tumorgraft (or patient-derived xenograft, PDX) model (Fig. 2). RCC tumorgrafts have shown promise as models in preclinical experimentation preserving the molecular genetics and biology of the corresponding patient tumor [9]. The patient’s tumor was particularly aggressive and a renal mass could be palpated as early as 18 days post-implantation, which is unusual [10]. After 83 days, the tumor had reached 1500 mm^3^ and was passaged to subsequent cohorts. Histological characterization of the tumorgraft revealed preservation of the morphology of the patient’s tumor, with extensive sarcomatoid differentiation and high levels of PD-L1 expression by IHC (Fig. 2a).

One month from initial staging scans, repeat computed tomography (CT) imaging revealed progression of lung and rib metastases. The patient enrolled in a clinical trial combining stereotactic ablative radiotherapy (SABR) and HD-IL2 [11]. He received SABR treatments to his left rib (25 Gy, one fraction) and a left lung metastasis (25 Gy, one fraction) followed by two courses of 600,000 international units/kg IV of HD-IL2 q 8 h. He received ten and nine doses of HD-IL2, two weeks apart. Subsequent imaging studies demonstrated improvement in the radiated lung and rib metastases (Fig. 1a). Otherwise, there was a mixed response with improvement in some non-radiated lung nodules, but also the development of new metastases in the lungs, lymph nodes, and right femur (Fig. 1b).

In June 2017, the patient was switched to pazopanib (800 mg PO qd). He also underwent a right total knee replacement followed by adjuvant radiation (20 Gy over 5 fractions). Repeat scans after three months demonstrated progression of existing lung and nodal metastases, and three new right shoulder metastases, which were painful (Fig. 1c).

One week later, the patient began nivolumab monotherapy (240 mg IV q 2 weeks). A few days after the initial infusion, the patient developed grade 2 dermatitis (by Common Terminology Criteria for Adverse Events, CTCAE) [12] at the sites of prior rib and knee radiation, possibly related to radiation recall (Fig. 1d). Inflammation improved over the following month during which nivolumab was withheld. Upon resolution, the patient resumed treatment with nivolumab and received three additional doses without a dermatologic reaction.

Restaging CT scans three months after the initial nivolumab infusion showed a decrease in size of all lung metastases (Fig. 1e), stable disease at all other sites and no new lesions. The patient continued nivolumab for five additional infusions with interim development of grade 2 hypothyroidism managed with levothyroxine therapy, but then stopped infusions after the development of autoimmune colitis (confirmed by colonoscopy and biopsy; Fig. 1f).

CT and magnetic resonance (MR) scans at this time, eight months from initial nivolumab infusion, confirmed an iRECIST [13] PR with an interval improvement in several sites including the shoulder metastases. Following resolution of the diarrhea, the patient received additional nivolumab but developed grade 3 autoimmune hepatitis requiring intravenous steroids and mycophenolate mofetil, and nivolumab was discontinued.

Seven months after the last infusion of nivolumab and 2 years since diagnosis, the patient remains off immunotherapy without progression and near complete resolution of shoulder metastases (Fig. 1e).

### Immuno-PET

We radiolabeled atezolizumab, a monoclonal anti-PD-L1 antibody with a mutant Fc, with zirconium-89 [^89^Zr]. Zirconium-89 is a well-studied positron emitting radioisotope used to label antibodies with a half-life of 78 h, which is compatible with the slower pharmacokinetics of antibodies [14]. This allows imaging to be performed for several days after injection to improve tumor-to-background signal. Accumulation of the isotope in tumor sites over time and clearance from other sites improves contrast.

Methodologically, the antibody was conjugated to the chelator deferoxamine (DFO) at a molar ratio of 1:1.9 and radiolabeled with ^89^Zr (5 mCi per mg of DFO-atezolizumab conjugate) using previously published protocols [15, 16]. Briefly, the DFO-atezolizumab conjugate was incubated with neutralized ^89^Zr for 1 h, and the reaction was quenched with 50 mM diethylenetriamine pentaacetic acid. The radiolabeled antibody fraction was purified using the Zeba™ centrifugal spin columns (40 K MWCO) and eluted in 0.2 M sodium acetate buffer containing 5 mg/mL gentisic acid (pH 5.5–5.6). The conjugate had a specific radioactivity of 2–4 mCi/mg protein, with high radiochemical purity (≥ 99%). The immunoreactivity of the radiolabeled immunoconjugate was confirmed using an in vitro cell-based Lindmo assay [17] and was 86.2 ± 4% (*n* = 6). In addition, the conjugate was tested for stability in plasma and was found to be quite stable (> 80% of ^89^Zr activity retained with atezolizumab in rat serum at 37 °C after 7 days).

Mice bearing the patient-derived tumorgrafts were injected intravenously (by tail-vein) with ~ 100 μCi of ^89^Zr-DFO-atezolizumab. A second tumorgraft line from a tumor expressing low levels of PD-L1 (< 1%) by IHC was chosen as a negative control (Fig. 2c). PD-L1 IHC procedures and interpretations were standardized (Biocare Medical, Clone ACI3171A,C; 1:300) and results were scored by a pathologist blinded to other results.

Mice were serially imaged on a Siemens Inveon PET/CT system. PET quantification was performed blinded. PET imaging at day 6 post-injection (d.p.i.) showed a statistically significant difference in ^89^Zr signal between the index patient tumorgrafts (4.2 ± 0.6% injected dose/g [%ID/g]; *n* = 3) and the controls (3.1 ± 0.5% ID/g; *n* = 3) (*p* = 0.028) (see Fig. 2). Similar results were observed with a second independent cohort of tumorgrafts (5.2 ± 0.4% ID/g; *n* = 3) compared to the same control group (*p* = 0.002).

Differences in tumor uptake could not be accounted for by differences in tumorgraft volumes, which were not significantly different between the index and control groups (831.9 ± 473 mm^3^ versus 1010.3 ± 492.6 mm^3^; *p* = 0.62, respectively). Further, the tumor/muscle contrast in the index tumorgrafts was 4.4 ± 0.4, which is also significantly higher than controls (2.7 ± 0.6) (*p* < 0.05). (All statistical analyses were performed using GraphPad Prism 7 by un-paired t-tests without correction for multiple comparisons and an alpha value of 0.05.) Following the last PET scan, mice were sacrificed, and tumorgrafts and other vital organs were collected for IHC assays. IHC analyses of the tumorgrafts confirmed expected levels of PD-L1 expression.

## Discussion and conclusion

We report a patient with metastatic ccRCC and sarcomatoid differentiation who had high-levels of PD-L1 expression in tumor cells and had a sustained response to nivolumab as well as preclinical studies using PD-L1 iPET in a corresponding tumorgraft. While there have been studies of ^89^Zr-labeled atezolizumab in other types of cancer [18], to our knowledge this is the first study to be reported in RCC. Another distinguishing feature of our study derives from being able to correlate the findings in the mouse model to those in the corresponding patient. Indeed, inasmuch as response rates in metastatic RCC to single agent nivolumab are around 25% [3], the development of molecular imaging tools to identify (or enrich) for these patients would be beneficial. We hypothesize that one factor may be PD-L1 expression. As such, iPET assays could be helpful. It is well established that antibody-based reagents such as trastuzumab or rituximab are highly specific and only effective against tumors that express the target. The same may be expected of anti-PD-L1 antibodies. Accordingly, tumors devoid of PD-L1 expression are unlikely to respond to anti-PD-L1 antibodies (or antibodies to the corresponding receptor, PD-1).

Serial iPET scans present the ability to monitor tumor PD-L1 expression over time, allowing dynamic assessment of therapeutic interventions. For instance, in our particular patient, PD-L1 expression levels might have been further increased by prior IL2 treatment. Such a finding would provide further justification to evaluate IL2 in combination with anti-PD-1/PD-L1 therapies. Conversely, one might observe loss of PD-L1 expression at some sites over time, which may harbinger the development of resistance.

Another feature of PD-L1 iPET is the evaluation of PD-L1 expression in non-tumor sites, which may help with predicting toxicity such as the irAEs seen in our patient. For example, radiation may upregulate PD-L1 expression in the tumor microenvironment [19, 20]. This effect may extend to native tissues such as keratinocytes, which upregulate PD-L1 expression when exposed to cytokines [21, 22]. These cytokines, including IFN-γ, are known to be released after radiation, and may predispose native tissue to T-cell-mediated attack upon checkpoint blockade. These series of observations are just one hypothesis for the pathogenesis of radiation recall dermatitis specific to checkpoint inhibitors, which was observed in our patient.

Our study has several limitations. First, while it provides a proof-of-principle, it represents a single case report with a control arm. Second, the iPET studies were performed in tumorgrafts, which do not capture the heterogeneity of patients’ tumors, nor the impact of therapies subsequent to their generation. Third, the discriminative ability of iPET remains to be fully determined. In addition, iPET will not be able to differentiate between PD-L1 expression in tumor vs. non-tumor cells at sites of metastases. Finally, the studies are performed in immunocompromised mice. Despite these caveats, a recent first-in-human study of ^89^Zr-atezolizumab iPET in 22 patients with metastatic bladder cancer, non-small cell lung cancer, or triple-negative breast cancer found a correlation of pre-treatment radiotracer uptake with both progression free and overall survival with atezolizumab, while conventional IHC staining of PD-L1 failed to meet significance in predicting benefit [18].

Assessing the potential of iPET in RCC will require studies in patients. We filed an Investigational New Drug (IND) application and obtained approval to study ^89^Zr-atezolizumab in patients at UT Southwestern.

## Abbreviations

% ID/g: Percent Injected Dose per gram
^89^Zr: Zirconium-89
ccRCC: clear cell Renal Cell Carcinoma
CT: Computed Tomography
d.p.i.: days post-injection
Fc: Fragment crystallizable
h: hours
HD-IL2: High-Dose Interleukin 2
IHC: Immunohistochemistry
IL2: Interleukin 2
IMDC: International Metastatic Database Consortium
IND: Investigational New Drug
iPET: immuno-PET
irAE: immune-related Adverse Event
ISUP: International Society of Urological Pathology
IV: Intravenous
MR: Magnetic Resonance
NOD/SCID: Non-Obese Diabetic with Severe Combined Immunodeficiency
PD-1: Programmed Cell Death Protein 1
PD-L1: Programmed Death-Ligand 1
PDX: Patient-Derived Xenograft
PET: Positron Emission Tomography
PO: Oral
PR: Partial Response
q: every
qd: every day
RCC: Renal Cell Carcinoma
SABR: Stereotactic Ablative Radiotherapy
TKI: Tyrosine Kinase Inhibitor
